# CRISPR/Cas9-Based Lateral Flow and Fluorescence Diagnostics

**DOI:** 10.3390/bioengineering8020023

**Published:** 2021-02-12

**Authors:** Mark J. Osborn, Akshay Bhardwaj, Samuel P. Bingea, Friederike Knipping, Colby J. Feser, Christopher J. Lees, Daniel P. Collins, Clifford J. Steer, Bruce R. Blazar, Jakub Tolar

**Affiliations:** 1 Department of Pediatrics, Division of Blood and Marrow Transplant & Cellular Therapy, University of Minnesota Medical School, Minneapolis, MN 55455, USA; bhard009@umn.edu (A.B.); binge042@umn.edu (S.P.B.); fknippin@umn.edu (F.K.); feser004@umn.edu (C.J.F.); leesx002@umn.edu (C.J.L.); blaza001@umn.edu (B.R.B.); tolar003@umn.edu (J.T.); 2 Cytomedical Design Group, LLC, Saint Paul, MN 55127, USA; dc@cmdgllc.com; 3 Department of Medicine, University of Minnesota Medical School, Minneapolis, MN 55455, USA; steer001@umn.edu; 4 Department of Genetics, Cell Biology and Development, University of Minnesota Medical School, Minneapolis, MN 55455, USA

**Keywords:** CRISPR/Cas9, SARS-Co-V2, lateral flow assay

## Abstract

Clustered regularly interspaced short palindromic repeat (CRISPR/Cas) proteins can be designed to bind specified DNA and RNA sequences and hold great promise for the accurate detection of nucleic acids for diagnostics. We integrated commercially available reagents into a CRISPR/Cas9-based lateral flow assay that can detect severe acute respiratory syndrome coronavirus 2 (SARS-CoV-2) sequences with single-base specificity. This approach requires minimal equipment and represents a simplified platform for field-based deployment. We also developed a rapid, multiplex fluorescence CRISPR/Cas9 nuclease cleavage assay capable of detecting and differentiating SARS-CoV-2, influenza A and B, and respiratory syncytial virus in a single reaction. Our findings provide proof-of-principle for CRISPR/Cas9 point-of-care diagnosis as well as a scalable fluorescent platform for identifying respiratory viral pathogens with overlapping symptomology.

## 1. Introduction

The World Health Organization declared a pandemic in March of 2020 as a result of the global spread of the severe acute respiratory syndrome coronavirus 2 (SARS-CoV-2) that causes coronavirus disease 2019 (COVID-19). Testing and contact tracing are cornerstones of prevention, mitigation, and control efforts. Patient demographics for those who succumb to COVID-19 are multifactorial; however, the ability to test at high capacity has been associated with reduced mortality [1]. The ability to test often correlates with better infrastructure, hospital capacity, healthcare quality, and effective public health systems. The ‘gold standard’ test for COVID-19 has been quantitative reverse transcription polymerase chain reaction (qRT-PCR) to detect viral nucleic acid and has been adopted by most public health agencies. Serological and antigen detection assays also comprise a minority of tests and are more streamlined with faster turnaround times. However, the nature of the immune response whereby IgM and IgG antibodies are produced can be days to weeks following exposure to the virus [2] and antigen testing is less sensitive, particularly for asymptomatic patient diagnoses [3,4]. Therefore, nucleic acid testing by qRT-PCR remains the most broadly applied procedure and requires sample collection, transport, RNA isolation, reverse transcription, amplification, sequence interrogation, and detection. Detection by qRT-PCR relies on a primer:probe set that specifically recognizes a target DNA sequence and generates a fluorescent signal during DNA amplification. This testing platform is based on specialized reagents, training, and infrastructure that is costly and can lead to turnaround times of >24 h. This longer test turnaround time can result in multiple exposures leading to unchecked virus spread.

Advances in rapid nucleic acid amplification and detection hold promise for streamlining diagnostics and shortening test turnaround times. Isothermal amplification using recombinase polymerase amplification (RPA) or loop-mediated isothermal amplification (LAMP) offer robust amplification without the need for temperature cycling or specialized equipment [5]. RPA is comprised of recombinase proteins that bind primers and facilitate binding to homologous sequences. Primer binding and strand displacement is stabilized by single-stranded binding proteins followed by recombinase dissociation that allows for polymerase binding to the primer and amplification [6]. LAMP uses primers for gene-specific amplification as well as two specialty primers that form loop structures that allow for multiple rounds of amplification [7]. Following, or in concert with, nucleic acid amplification, enzymes from the clustered regularly interspaced short palindromic repeats (CRISPR) platform have been employed for detection/diagnostics. CRISPR/Cas12 and Cas13 have nucleic acid cleavage abilities such that target nucleic acid interrogation and recognition leads to ectopic strand breaks of a secondary reporter molecule that can be detected by lateral flow assays (LFA) or fluorometry [8,9,10,11,12,13]. CRISPR/Cas9 has also been developed for diagnostics using solid-state nanopores, microfluidics, and LFA [14,15,16,17,18]. Some of these Cas9-based methodologies can be cumbersome to employ and can require specialized starting material, formulations, or instrumentation [14,15,18,19,20,21,22,23,24]. Wang and colleagues streamlined the application of CRISPR/Cas9 for LFA by using a novel gold nanoparticle (Au-NP) probe that was designed to bind a Cas9 conjugated guide RNA (gRNA) [18]. We sought to extend this concept by designing a fully commercialized reagent approach in order to avoid specialized design requirements or reagent generation. The LFA test strips employed bind fluorescein isothiocyanate (FITC)/6-Carboxyfluorescein (FAM) and biotin to generate a positive result. Therefore, we used a FITC/FAM-labeled PCR primer and a nuclease inactive (‘dead’) biotinylated Cas9 and a single guide RNA (sgRNA) specific for the ORF8a gene of SARS-Co-V-2 to label amplicons for detection by LFA. This approach was capable of single-nucleotide resolution and avoided false positives from primer dimer or non-specific amplification artifacts that can occur with the use of tandem FITC- and biotin-labeled primers for LFA [25].

Other studies have employed the nuclease properties of Cas proteins to generate fluorescence signals upon encountering target nucleic acid(s) [8,9,10,11,12,13,26,27,28]. We built off these principles by developing a sensitive fluorescence-based assay to detect the SARS-CoV-2 ORF8a gene sequence. This concept relied on a DNA probe with a quenched fluorophore to bind the target and be cleaved by Cas9. Further, the multiplex abilities of CRISPR/Cas9 showed the ability to both detect and distinguish between respiratory viruses that can exhibit similar physical symptoms. With these defined and optimized LFA and fluorescence detection strategies, we validated their diagnostic potential in a coronaviral genomic RNA isolate.

Overall, the LFA procedure promotes field-based diagnostic capabilities with low cost and little need for special infrastructure. The fluorescence methodology allows for specific, scalable detection of SARS-CoV-2, influenza A and B, and/or respiratory syncytial virus (RSV) in a multiplex fashion.

## 2. Materials and Methods

### 2.1. Nucleic Acids

Oligonucleotide and sgRNAs are detailed by sequence and function in Table 1, Table 2, Table 3 and Table 4. All primers and synthetic fragments were produced by Integrated DNA Technologies (IDT), Coralville, IA, and are shown 5′-3′. 

### 2.2. Amplification

DNA template fragments were amplified with the indicated forward and reverse primers.

Endpoint PCR was performed using 1 µM template and 0.2 µM final concentration of primers in a 100 µL reaction volume with Phusion High-Fidelity PCR Master Mix (Thermo Fisher, Waltham, MA) under the following conditions: 98 °C × 2 min and 34 cycles of 98 °C × 10 s, 62 °C × 10 s, and 72 °C × 15 s, with a final extension of 5 min.

Recombinase polymerase amplification (RPA): 1 µM of template was used with primers for recombinase polymerase amplification according to the manufacturer’s instructions for the TwistDx Basic RPA Kit from TwistDx (Maidenhead, UK).

Fluorescent Probes: Probes were purchased from IDT (Coralville, IA, USA) and resuspended at 100 µM and contain Iowa Black quencher (3IABkFQ or 3IAbRQSp). The 5′ fluorescent labels are: FAM = 6-Carboxyfluorescein, TexRd = Texas Red, YakYel = Yakima Yellow, Cy3 = Cyanine 3, and TAMRA = 5-Carboxytetramethylrhodamine.

### 2.3. Soak DNA Oligonucleotides

The oligonucleotides were purchased from IDT (Coralville, IA, USA) and resuspended at 200 µM. Equal molar equivalents were mixed in Tris NaCl and denatured and renatured by heating for 5 min at 95 °C and cooling to room temperature at a rate of −0.1 °C/s.

### 2.4. Guide RNAs

All single guide RNAs (sgRNA) were purchased from Synthego (Menlo Park, CA, USA) and resuspended at 100 µM in Tris Ethylenediaminetetraacetic acid. Shown are the 20 bp sequences specific to the corresponding gene target. The remaining sgRNA architecture is the vendor supplied standard sequence for *Streptococcus pyogenes* Cas9 binding.

### 2.5. Biotinylated Cas9 and Lateral Flow

dCas9-3XFLAG™-Biotin Protein containing the D10A and H840A mutations from Milipore Sigma (Merck KGaA, Darmstadt, Germany) was resuspended in the included Reconstitution Solution to ~1.7 mg/mL (8 pmol/µL), and 1 µL of 100 µM sgRNA was added to give ~1.2 M excess of sgRNA. Cas9:sgRNA complexing was allowed to occur at room temperature for five minutes.

10 µL of unpurified PCR or RPA products was used with the Cas9:sgRNA complex in a 50 µL reaction at 37 °C. Where indicated, the double-stranded soak DNAs were used at the concentrations shown in the relevant figures. 20 µL of the Cas9 reaction was used for detection using the TwistDx Milenia HybriDetect1 lateral flow assay (Maidenhead, UK) under the manufacturer’s recommendation.

For the assays in which Cas9 was included in the RPA, the reaction conditions and primer concentrations remained as above. Instead of using water to achieve the final 50 µL reaction volume, the indicated concentrations of soak ODNs were used to reach the final volume. RPA then proceeded at 37 °C with use of 20 µL of the post-reaction product for LFA.

### 2.6. CRISPR/Cas9 Fluorescence Assay

Equal volumes of amplification product and 100 µM disease-specific probe were mixed and denatured/renatured by heating for 5 min at 95 °C and cooling to room temperature at a rate of −0.1 °C /second. 20 µL of this product was used for single or multiplex fluorescence with 10 µg of Cas9 nuclease (Aldevron, Fargo, ND, USA) and 100 µM of sgRNA in 100 µL at 1X New England BioLabs buffer 3.1 (New England BioLabs, Ipswich, MA, USA).

Single fluorescence assays were performed in a black 96-well plate (ThermoFisher, Waltham, MA, USA) and signal was recorded using excitation: 485 nm, emission: 530 on a BioTek (Winooski, VT) plate reader. Multiplex fluorescence was performed in a 96-well skirted PCR plate (ThermoFisher, Waltham, MA, USA) and fluorescence was recorded in the FAM, VIC®, TAMRA (Carboxytetramethylrhodamine), and JUN™ channels every 30 s over one hour using the QuantStudio3 Real-Time PCR System (ThermoFisher, Waltham, MA, USA).

### 2.7. RT-PCR

Genomic RNA from severe acute respiratory syndrome-related coronavirus 2 (ATCC^®^ VR-1986D™) was acquired from the American Type Culture Collection (Baltimore, MD) and was deposited by the Centers for Disease Control and Prevention and obtained through BEI Resources, NIAID, NIH (Biodefense and Emerging Infections Research Resources Repository, National Institute of Allergy and Infectious Diseases, National Institutes of Health) Genomic RNA from SARS-Related Coronavirus 2, Isolate USA-WA1/2020, NR-52285.

Reverse transcription was performed with SuperScript™ IV Reverse Transcriptase Master Mix (ThermoFisher, Waltham, MA, USA).

Real-time PCR of SARS-Co-V2 cDNA was performed with the 2019-nCoV RUO Kit (IDT, Coralville, IA) following the manufacturer’s recommendations and using the QuantStudio3 Real-Time PCR System (ThermoFisher, Waltham, MA, USA). Human glyceraldehyde 3-phosphate dehydrogenase (*GAPDH*) was detected with the Hs02786624_g1 probe (ThermoFisher, Waltham, MA, USA).

### 2.8. Genome Analysis

SARS-CoV-2 genome sequence was obtained from the global initiative on sharing avian influenza data current to January 2021 (www.gisaid.org (accessed on 9 February 2021)).

### 2.9. Graphing and Statistics

Values were graphed using GraphPad Prism 9 (San Diego, CA, USA) and statistical evaluation was performed using one-way analysis of variance (ANOVA) and a post hoc Tukey’s multiple comparisons test.

### 2.10. Images

Photography was performed with a Canon 5DIII with a Tamron 25–70 lens at the 70 mm setting. Figure images were produced with BioRender.com (Toronto, ON, Canada).

## 3. Results

### 3.1. Rapid Nucleic Amplification and Lateral Flow Detection

**Figure 1:**
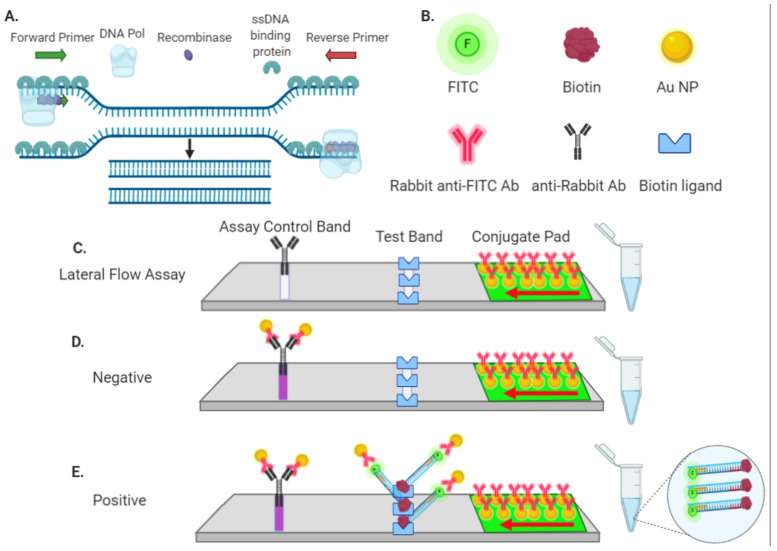
Recombinase polymerase amplification (RPA) and nucleic acid detection by lateral flow assay. (**A**) Recombinase polymerase amplification. Recombinase proteins complex with primers and facilitate strand displacement and binding to homologous target sequence(s) without requiring temperature alterations (i.e., cycling). The DNA polymerase initiates synthesis from the primers and amplifies target DNA exponentially. (**B**–**E**) Analyte detection by lateral flow assay (LFA). (**B**) Reagent components for detection via LFA (Au-NP = gold nanoparticle, Ab = antibody, FITC = fluorescein isothiocyanate). (**C**) Embedded in the flow assay device conjugate pad are gold nanoparticles decorated with rabbit anti-FITC antibodies. The test and assay control bands are coated with a biotin ligand or anti-rabbit antibodies, respectively. A red arrow indicates the direction of sample flow through the conjugation pad. In the absence of a molecule that is labeled by both FITC and biotin, the Au-NPs flow to and accumulate at the assay control band, where they are bound by anti-rabbit antibodies. Dual FITC:biotin-labeled substrates are bound first by the anti-FITC Au-NPs and then accumulate at the test band via capture of the biotin label by its ligand. Because Au-NPs are in excess, Au-NPs containing only the rabbit-anti-FITC Ab also flow to the assay control band. Accumulated Au-NPs are observed, and results are interpreted as (**D**) negative when only an assay control band is present or (**E**) positive with both test and assay control bands are visible.

**Figure 2:**
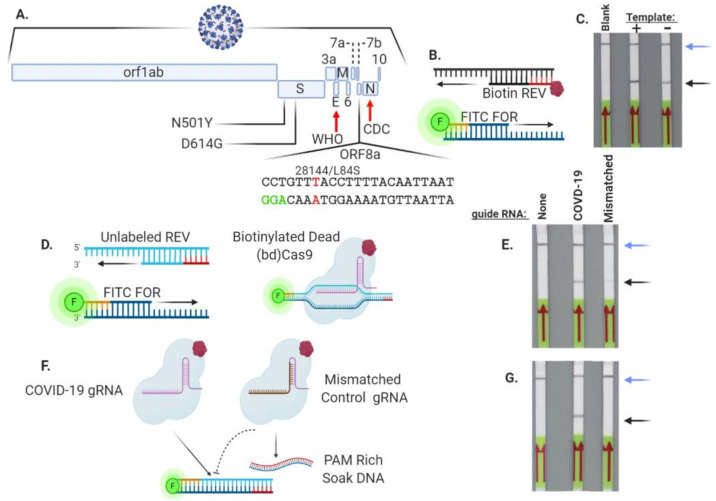
Biotinylated ‘dead’ Cas9 (bdCas9) and PAM-rich competitor soak DNA allows for the detection of target nucleic acids. (**A**) SARS-CoV-2 genome and detection strategy. The viral genes are shown as well as the D614G and N501Y amino acid mutations in the S gene and the L84S alteration in ORF8a. The locations of the WHO and CDC genes analyzed in their respective diagnostic assays are indicated with red arrows. The sequence in the ORF8a gene targeted for CRISPR/Cas9 sgRNA detection in the present study is shown with the L84S polymorphic nucleotide at position 28144 (indicated in red) and the CRISPR/Cas9 protospacer adjacent motif (highlighted in green). (**B**, **C**) Dual-labeled PCR primer strategy results in false positives. Amplification was performed with a FITC-labeled forward and biotinylated reverse primer in the absence (−) or presence (+) of a template, and products were analyzed via LFA (**C**). ’Blank’ refers to an LFA test strip loaded with no amplicon. (**D**, **E**) Uncoupling the FITC and biotin labeling of DNA using a FITC primer and bdCas9 allows for nucleic acid detection. Amplification was performed with a FITC-labeled forward and unlabeled reverse primer. The amplicon was then interrogated with a SARS-CoV-2-specific or mismatched control sgRNA. (**E**) The presence of test bands in samples with either sgRNA demonstrates non-specific dCas9 binding of target DNA. (**F**, **G**) A competing PAM-rich soak double-stranded oligonucleotide prevents non-specific bdCas9/mismatched sgRNA binding. (**F**) A double-stranded oligonucleotide rich in GGG trinucleotide PAMs was included in the reaction mixture containing FITC-labeled COVID DNA amplicons and a COVID sgRNA or an unmatched control sgRNA. (**G**) LFA of COVID-19 amplicons with COVID-19 or control/mismatched sgRNA LFA detection shows specificity in the presence of the competitor soak DNA. Blue and black arrows in (**C**), (**E**), and (**G**) designate the assay control and test bands on the LFA, respectively. Results are representative of at least four independent experiments.

Isothermal RPA amplification and detection via LFA are a simplified approach for nucleic acid analysis that avoids the need for specialized infrastructure (Figure 1A) [6,25]. The LFA strips employed in our study require test material that is labeled with both FITC/FAM and biotin (Figure 1B–E). FITC/FAM:biotin conjugated analytes are captured at the test band that contains a biotin ligand (Figure 1C,E). FITC Au-NPs are in excess and a portion remain unbound and flow to the assay control band (Figure 1D). This ensures that the LFA test strip is functional and suitable for interpretation. We designed forward and reverse PCR primers that were labeled with FITC and biotin respectively, to generate SARS-CoV-2 ORF8a gene amplicons that flanked a single nucleotide polymorphism at genomic nucleotide position 28144 that causes a L84S amino acid substitution (Figure 2A,B). ORF8a is a ~100 amino acid protein with putative ER import signal sequences [29]. While the Centers for Disease Control and Prevention (CDC) and World Health Organization (WHO) assays detect the N and E genes respectively, we designed our assay for the ORF8a region of the SARS-CoV-2 genome because it does not overlap with other viral sequences and the L84S alteration was the first mutation observed in the USA (Figure 2A) [30]. This mutation is proximal to a protospacer-adjacent motif (PAM) of –NGG (N = any nucleotide and G = guanine) for the *Streptococcus pyogenes* Cas9, making it suited for Cas9 detection (Figure 2A). The use of dual-labeled (i.e., FAM and biotin) primers resulted in positive LFA test bands in either the presence or absence of an ORF8a template (Figure 2C). We hypothesized that primer dimers that resulted in a single complex containing FITC and biotin were the cause of this false positive result and corrected it by titrating the primer concentration. It was observed that higher primer concentrations contributed to dimerization and high false positive rates and lower primer amounts diminished sensitivity (Figure S1, in the Supplementary Materials). These results showed that direct amplification with labeled primers may be suboptimal for unambiguous nucleic acid detection of ORF8a by LFA.

### 3.2. Cas9 Allows for Specific SARS-Co-V2 ORF8a Sequence Detection

In order to avoid prohibitively high false positive test results, the labeling of the target amplicon was next approached using a FAM/FITC forward primer and an unlabeled reverse primer (Figure 2D). We posited that biotinylation of this FITC-labeled PCR product could be accomplished using a biotinylated, nuclease inactivated (‘dead’) version of Cas9 (bdCas9) (Figure 2D). FITC ORF8a amplicons generated by standard PCR were incubated with bdCas9 and an ORF8a-specific sgRNA (Figure 2A) or a mismatched control sgRNA (Supplementary Figure S2A). Under these conditions, we observed a readily observable test band using the COVID-19 sgRNA and a faint one using the control sgRNA with no homology to SARS-Co-V2 (Figure 2E). Densitometry has been applied to ascribe values for semi-quantitative results as well as to differentiate the test bands of experimental and controls [31]. Because Cas9 is physically associated with DNA while scanning for target sites to cleave [32], we predicted that this interaction led to non-specific DNA labeling and a faint but visible test band in the control sgRNA (Figure 2E). Therefore, to improve definitive interpretation, a competing ‘soak’ DNA that was rich in PAM-NGG sequences was designed in order to prevent indiscriminate Cas9:DNA interactions (Figure 2F and Figure S2B). This allowed for specific detection using a COVID-19 sgRNA, while the control mismatched sgRNA did not show a test band, therefore false positive detection was avoided (Figure 2G). Conversely, an irrelevant amplicon was not recognized by the COVID-19 sgRNA (Supplementary Figure S2C). These results confirmed the ability of bdCas9 to label a FITC amplicon for detection via LFA.

**Figure 3:**
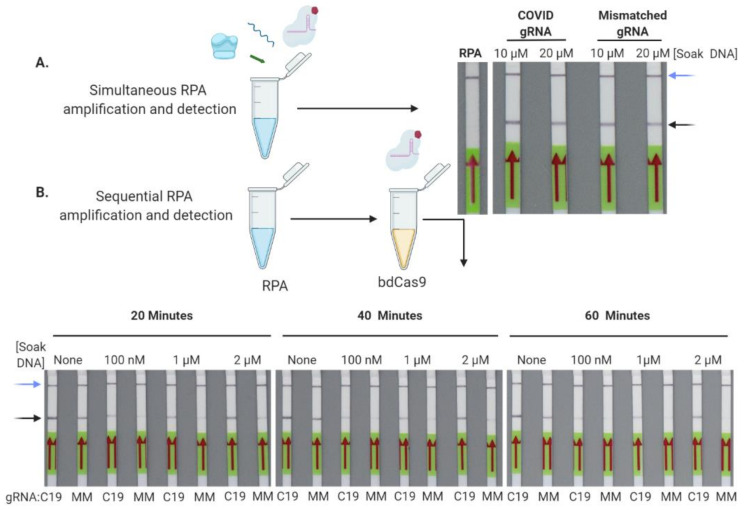
Optimization of rapid SARS-Co-V2 nucleic acid detection. (**A**) Simultaneous RPA and bdCas9 detection results in false positives. Room temperature RPA of a SARS-CoV-2 template was performed in the presence of bdCas9 plus 10 or 20 µM competitor (i.e., PAM) soak DNA with a COVID-19 or mismatched sgRNA and analyzed by LFA. (**B**) Sequential RPA and Cas9 detection. RPA was performed at room temperature followed by amplicon incubation with bdCas9. RPA products were incubated with the indicated concentrations of competitor PAM soak DNA for 20, 40, or 60 min with either a COVID-19 (C19) or mismatched (MM) irrelevant sgRNA, followed by LFA. Blue arrows show the assay control band and black arrows indicate a test band. Images are representative of three independent experiments. LFA-labeled RPA in (**A**) represents a no DNA template control.

To merge the capabilities of RPA and our bdCas9-based detection method, we investigated the conditions for rapid SARS-Co-V2 sequence detection. First, RPA using a FITC-labeled forward and unlabeled reverse primer was performed in the presence of bdCas9. Even with a large excess of competitor soak DNA, a positive test band was observed on LFA strips with either COVID or control sgRNAs when a SARS-Co-V2 DNA template was present (Figure 3A). The two reaction components were then separated, first by performing a 20 min RPA at room temperature and then using RPA products for bdCas9 detection. With a 20 min bdCas9 incubation, test bands were visible that became more defined at 40 and 60 min and with increasing amounts of soak DNA (Figure 3B). Collectively, these data showed that SARS-CoV-2 DNA amplified by RPA can be detected with bdCas9 and LFA in the presence of a competitor/soak DNA.

### 3.3. Cas9-Nuclease-Based Diagnostics for Single and Multiplexed Targets

**Figure 4:**
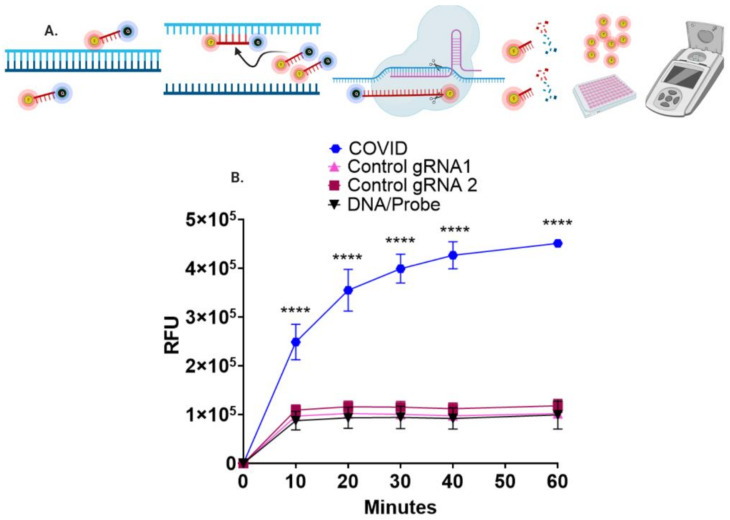
Fluorescence-based CRISPR/Cas9 nuclease detection of SARS-CoV-2 DNA. (**A**) Design of a CRISPR/Cas9 nuclease fluorescence detection assay. SARS-CoV-2 DNA amplicons were mixed with DNA probes containing a 5′ fluorescein fluorophore and 3′ Iowa black quencher. The probe and amplicons were denatured and renatured, resulting in a heteroduplex of amplicon:probe DNA that was incubated with CRISPR/Cas9 nuclease complexed with COVID-19, or control sgRNAs. Cas9 nucleolytic activity uncouples FAM from the quencher, resulting in a fluorescent signal that can be measured with a fluorometer. (**B**) CRISPR/Cas9 nuclease fluorescent signal detection of SARS-CoV-2 DNA. A time course was performed to evaluate fluorescence signal intensity generated by Cas9 nuclease conjugated with a COVID-19 sgRNA, two separate unmatched control sgRNAs, or hybridized PCR:probe duplexes (DNA/probe) alone. Shown is the mean relative fluorescent value (RFU) and standard deviation from four independent experiments. *p*-value (one-way analysis of variance (ANOVA) and Tukey’s multiple comparisons test) of **** <0.0001 is shown with asterisks.

**Figure 5:**
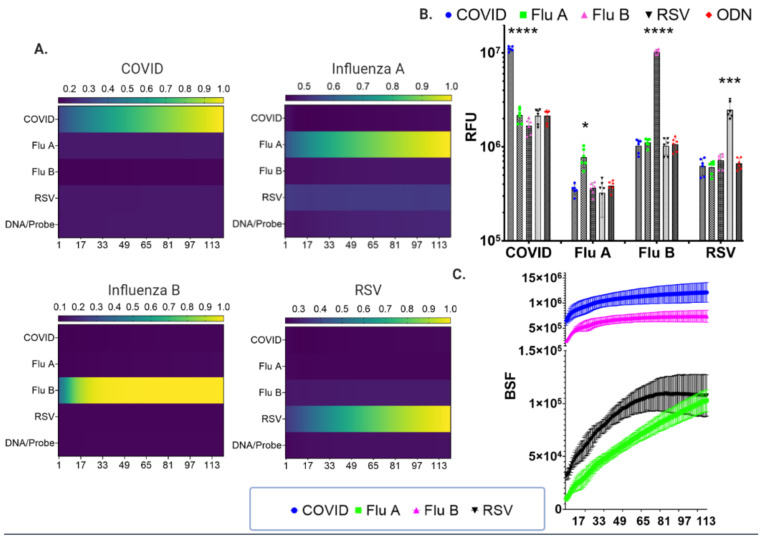
Multiplex CRISPR/Cas9 fluorescence detection of viral respiratory pathogens. (**A**) CRISPR/Cas9 shows high specificity in distinguishing respiratory pathogen targets. Individual DNA amplicons and fluorescent probes for SARS-CoV-2 (FAM-labeled), influenza A (Flu A; TxRed^®^-labeled) or B (Flu B; Yakima Yellow^®^-labeled), or respiratory syncytial virus (RSV; TAMRA-labeled) were annealed and interrogated with Cas9 and sgRNAs for each virus DNA. The DNA target amplicons are indicated at the top of each graph and the sgRNAs are labeled vertically on the y-axis. The heat maps represent the mean fluorescent values from three independent experiments performed in duplicate. The horizontal color bars at the top of each heat map are the values normalized to the highest fluorescent signal obtained. (**B**) Fluorescence values are plotted as the mean of the three independent experiments performed in duplicate from (**A**). Statistical evaluation of these data was done using one-way ANOVA and Tukey’s multiple comparison to show fluorescence signal above other analytes. *, ***, *** indicates *p* = <0.05, *p* = <0.001, and *p* = <0.0001 respectively, for the appropriate matched sgRNA versus the highest corresponding fluorescent value for an unmatched sgRNA. The y-axis is relative fluorescent units (RFU), the x-axis represents the DNA target, and the label at the top represents the sgRNA for COVID, Influenza A or B, RSV, and template:probe hybrids alone (ODN). (**C**) Multiplex detection of four respiratory viral pathogen sequences. Annealed probe:DNA complexes for each target were pooled and interrogated simultaneously with all four sgRNAs complexed with Cas9. Background subtracted fluorescent units (BFU) obtained by subtracting the fluorescence of controls (annealed DNA/Probe without Cas9) are shown as the mean and standard deviation of three experiments. The color-coded bars in the graph correspond to the color-coded identifiers at the left. The fluorescent values were acquired using a real-time PCR instrument that measured fluorescence signal every 30 s (1 cycle = 30 s for 121 cycles). The x-axes for the graphs in (**A**) and (**C**) are labeled as ‘cycles’ which represents the time at which fluorescence images were captured during the one hour 37 °C isothermal assay (1 cycle = 30 s).

The nuclease properties of Cas9 also hold potential to serve as a diagnostic platform by cleaving a fluorescent probe in a sequence-specific manner (Figure 4A). COVID-19-specific probes labeled with a fluorescent marker and quencher were designed, that when hybridized with SARS-CoV-2 amplicons, were cleaved by Cas9 nuclease together with a COVID-19 sgRNA (Figure 4B). The ability to multiplex Cas9 with multiple sgRNAs also allowed us to test whether we could achieve simultaneous detection of viruses with overlapping symptomology. First, we designed, built, and tested DNA probes with distinct fluorophores for SARS-CoV-2, influenza A and B, and RSV, respectively. These were tested and showed specificity of fluorescent signaling only for matched sgRNAs (Figure 5A,B). Next, the four distinct viral detection components were all combined in a single reaction mixture and analyzed simultaneously under isothermal (37 °C) conditions in a single tube using real-time fluorometry with a standard quantitative PCR instrument in a 96-well format (Figure 5C). Distinct fluorescence signals from cleavage of the pathogen-specific probe by the disease-specific sgRNA were observed. These results showed that all four viral pathogens could be detected in a multiplex fashion.

### 3.4. LFA and Fluorescence Assay Validation with SARS-CoV-2 Genomic RNA

**Figure 6:**
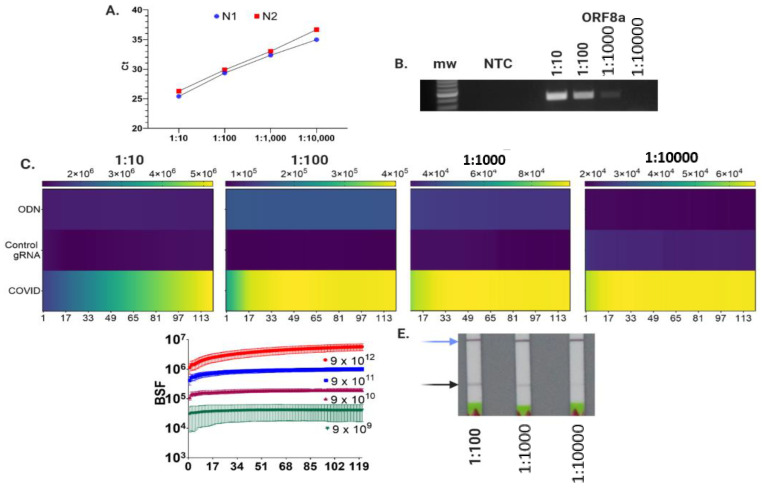
SARS-CoV-2 genomic RNA detection by LFA and fluorescence. (**A**) Viral RNA was diluted serially from 1:10 to 1:10,000 and reverse transcribed and analyzed by qRT-PCR using the CDC N1 and N2 primer:probe sets. Shown are triplicate samples of the mean ± standard deviation (SD) cycle threshold (Ct). (**B**) Viral cDNA was amplified as in (**A**) using ORF8a primers and analyzed by agarose gel electrophoresis. Mw = molecular weight and NTC = no template control. (**C**) Fluorescence detection of SARS-CoV-2 DNA targets. PCR products from serially diluted viral cDNA was hybridized with a COVID-19 probe and incubated with a Cas9:control sgRNA or COVID sgRNA and analyzed for fluorescence generation under isothermal (37 °C) conditions for one hour. Heat maps are shown of the mean of four experiments with the dilution series indicated at the top. Raw fluorescence value scales are shown at the top and the y-axis is the sample identification of DNA:probe alone (ODN) and DNA:probe hybrids interrogated with a control or COVID sgRNA. The x-axis represents the thirty second time points at which fluorescence was measured over the course of one hour (121 total measurements). (**D**) Copy number limit of detection (LOD). DNA obtained via PCR of SARS-CoV-2 reverse transcribed RNA was quantified and serially diluted to test the limit of detection using Cas9 nuclease generated fluorescence products. DNA:probe without Cas9 fluorescence values were subtracted and the y-axis represents these values as background subtracted fluorescence units (BFU). Values were measured every thirty seconds as in (**C**). Data are the mean values from three experiments. (**E**) Serially diluted ORF8a amplicons from viral cDNA were analyzed by LFA.

Genomic RNA from the USA-WA1/2020 isolate was diluted and reverse transcribed followed by qRT PCR with the designated primer:probes employed by the Centers for Disease Control and Prevention for the N gene (Figure 2A and Figure 6A, and Supplementary Figure S3). Using ORF8a primers, the same dilution series was performed and resolved by gel electrophoresis (Figure 6B). This amplicon was then used for establishing the limit of detection (LOD) with the fluorescence and LFAs. Fluorescence signal above background was observed for each sample, indicating that the Cas9-based assay limit of detection (LOD) was similar to that of qRT-PCR (Figure 6A,C). We further quantified ORF8a amplicons and performed a copy number LOD. Fluorescence above background was observed with 9 × 10^9^ copies of target DNA (Figure 6D). Under the parameters of 35 cycle exponential PCR amplification (2^35^), this represents <5 copies of starting material. LFA optimization with soak DNA and irrelevant sgRNA was performed (Supplementary Figure S4). Under these conditions, test bands were observable at a sensitivity that was an order of magnitude below that of qRT-PCR or Cas9 fluorescence (Figure 6E). Together, these results show that LFA and Cas9 fluorescence can be used for SARS-CoV-2 viral nucleic acid detection.

### 3.5. Cas9 Analysis of a SARS-CoV-2 Variant

Amino acid substitutions D614G and N501Y in the S gene and L84S in ORF8a have been suggested to result in increased viral load [33,34]. S gene D614G is highly prevalent [35] and N501Y has led to COVID surges [36]. Being able to distinguish SARS-CoV-2 strains may aid in whether certain strains are associated with differential clinical outcomes and/or could provide rapid information to public health departments. However, because the S gene between coronaviruses are highly homologous, we avoided targeting it with CRISPR/Cas9 to avoid false positives that may occur from a coronavirus other than SARS Co-V2. Instead, we targeted L84S caused by a SNP in the ORF8a gene that is unique to SARS-Co-V2 and delineated the relationships between S gene D614G, N501Y, and ORF8a L84S (Figure 2A and Figure S5). We tested the ability of Cas9 nuclease and bdCas9 to distinguish between cytosine and thymine at nucleotide position 28144 in ORF8a (Figure 2A). We interrogated the sequence of an ORF8a DNA amplicon with a perfectly matched fluorescent probe and sgRNAs with matched complementarity (thymine) or a one base pair mismatch (cytosine). Either wild-type or a high-fidelity version of Cas9 (SpyFi™) [37] were employed, and each showed differential fluorescence signal between matched and mismatched; however, it was not statistically significant (Figure S6A,B, in the Supplementary Materials). We then assessed the ability of bdCas9 in the LFA assay to distinguish targets at the single nucleotide level. For this, we utilized two soak DNA candidates: the PAM-rich soak DNA or a more homologous competitor soak that differed from the target and sgRNA by a single nucleotide (Figures S2B and S6C, in the Supplementary Materials). A test band was observed for both the perfectly matched and one base pair mismatched DNA target when the PAM-rich soak was used (Figure S6D,E, in the Supplementary Materials). In contrast, when using soak DNA containing the single nucleotide mismatch from the target, only the sgRNA with perfect match to the target yielded a test band (Figure S6D,E). These data showed that, using an appropriate soak DNA sequence, bdCas9 and LFA could resolve DNA targets at the single nucleotide level.

## 4. Discussion

The granting of emergency use authorization of SARS-Co-V2 vaccines represents a hopeful end to a pandemic that has infected more than 100 million people worldwide and claimed greater than 2 million lives from January 2020 to January 2021. It is predicted that widespread vaccine administration will not be available until the second or third quarter of 2021, making continued testing and mitigation efforts crucial to minimize more loss of life and continued global social, economic, and in-person schooling disruptions.

We set out to leverage the ability and specificity of *Streptococcus pyogenes* Cas9 to interrogate and identify SARS-Co-V2 sequences to develop testing platforms for both field-based and more specialized laboratory testing. The former requires simplified methodologies and rapid readouts and would be particularly helpful in rural areas that lack laboratory facilities able to perform molecular diagnostics. Rural COVID-19 case rates are increasing [38], rural residents are at elevated risk of COVID-19-related serious illness [39], medical care capacity in lowly populated areas can be quickly overwhelmed [40], and testing is challenging due to a lack of local facilities and funding [41]. To address these testing shortfalls, we employed Cas9 for targeting a portion of the SARS-CoV-2 ORF8a gene (Figure 2A). Presently, the CDC and WHO test for sequences in the N and E genes, respectively (Figure 2A). We chose ORF8a for our targeting strategies because there are seven coronaviruses that infect humans [42] and the ORF8a sequence is dissimilar between them, making it an ideal gene to target for SARS-CoV-2 detection without false positives occurring from these other viruses. Further, this site was ideal for designing a CRISPR/Cas9 sgRNA with an –NGG PAM (Figure 2A). The LFA test strips employed in our study require dual labeling of a candidate molecule for detection that is based on capture of the analyte by embedded Au-NPs coated with rabbit anti-FITC antibodies (Figure 1B,C). If the test material lacks a biotin label, it flows past the test band that contains a biotin ligand and accumulates at the assay control band that is coated with anti-rabbit antibodies. The presence of this band, that we termed the assay control, ensures that the LFA reagents and test stick device are functioning properly for detection (Figure 1D). If the reaction components are labeled with both FITC and biotin, a positive result is observed following Au-NP accumulation at the test band (Figure 1E). Using recombinase polymerase amplification (RPA) that allows for isothermal amplification of nucleic acids, we observed that the use of PCR primers labeled with FITC and biotin resulted in false positive test bands as a result of primer dimerization (Figure 2B,C and Supplementary Figure S1). In order to prevent this, we employed an amplification strategy using one FITC/FAM-labeled and one unlabeled primer (Figure 2D). To achieve the requisite FITC and biotin conjugation for LFA detection, we employed a nuclease inactive, biotinylated Cas9 with SARS-CoV-2 (COVID-19) or control sgRNAs. Under these conditions, we avoided the false positives resulting from primer dimers; however, control sgRNA with no homology to ORF8a showed the presence of a test band (Figure 2E). Cas9 can remain stably bound to DNA as it scans for sequences of homology required to initiate DNA cleavage [43]. In the absence of nucleolytic properties, such as with ‘dead’ Cas9, DNA is also scanned and the on/off rate is rapid and impacted by Cas9 concentration [44]. Therefore, the false positive test bands we observed may be due to high concentration of Cas9 that can associate and dissociate with the former, leading to test bands irrespective of the sgRNA. To remedy this, a bait or “soak” DNA sequence comprised of PAM-rich sequences was designed for inclusion in the assay in order to sequester non-specific binding events (Figure 2F and Supplementary Figure S2). Under these new conditions, LFA test bands were observed using the COVID-19 but not the mismatched control sgRNA (Figure 2G).

To further define the conditions for field-based testing, we explored the optimal settings for rapid amplification and detection via LFA. Previous studies using Cas enzymes have employed LAMP PCR and Cas in a ‘one pot’ approach for simultaneous amplification and detection [8,45]. In our system, this strategy yielded high levels of false positives even in the presence of large amounts of competitor soak DNA (Figure 3A). In order to prevent this, isothermal RPA was performed followed by biotinylated Cas9 interrogation. Increased amounts of soak DNA and longer dbCas9:sgRNA:DNA incubation times from 20 to 60 min improved the resolution of detection (Figure 3B). Centralized reference and public health diagnostic sites can still face backlogs, leading to increased turnaround time for results. Therefore, we sought to apply CRISPR/Cas9 toward developing a higher throughput methodology. The design was based on the nuclease properties of Cas9 to cleave a fluorescent probe hybridized to a target amplicon (Figure 4A). Following a 20 min isothermal room temperature RPA, a fluorescently labeled SARS-CoV-2 probe was annealed to the reaction product and incubated with Cas9 peptide and an ORF8a sgRNA. This resulted in rapid fluorescence generation with a statistically significant difference between Cas9 with COVID-19 sgRNA and control and DNA:probe reactions observable in as little as 10 minutes (Figure 4B).

COVID-19 symptoms can mirror those of influenza, which is most prevalent during the winter months in the northern hemisphere, and it is possible for one infection to be confused for another or for co-infection with both agents to occur [46]. Therefore, the CDC has designed multiplex qRT-PCR assays capable of detecting multiple viral pathogens in the same sample [47]. We likewise assessed the multiplex capability of Cas9 to detect and distinguish SARS-CoV-2, influenza A and B, and further designed and added components for detecting RSV. Individual sgRNAs were first tested against specific pathogen DNA:probe hybrids to assess whether there was any cross-reactivity. Figure 5A shows that only the Cas9:sgRNA complex matched to the target DNA:probe generated a distinct fluorescent signal. These data and those of Figure 2C,E are complementary and support the mechanism of Cas that interacts with and scans DNA in a broad manner but cleaves DNA in a sequence-specific fashion [48]. As such, in the bdCas9 binding assay, a competitor soak DNA is required to inhibit this process in order to achieve specificity by LFA, while the nuclease-dependent fluorescent approach is capable of specificity without competitor soak DNA (Figure 2 and Figure 5A). The detection kinetics for each target were distinguishable from mismatched sgRNA sample fluorescence signal (Figure 5B). Because no promiscuous activity was observed between any sgRNA, the amplicon:probe hybrids were then pooled for single-well multiplex analysis. Rapid fluorescence was generated that was readily distinguishable from controls (Figure 5C), showing the potential of CRISPR/Cas9 nuclease-based diagnostics. Importantly, this method can be performed on a quantitative PCR instrument, and while this represents specialized equipment, it is also standard equipment in many research laboratories and most public health facilities. The speed of detection, capability to multiplex, and the ability to perform reactions in a 96-well format (or greater) makes this a scalable platform for high-throughput analytics.

With proof-of-principle established for our methodology using synthetic fragments, we then validated both LFA and fluorescence detection using the USA-WA1/2020 coronaviral isolate. A dilution series was performed using the CDC qRT PCR assay with the N1 and N2 primer:probe set and the 1:10,000 dilution showed a Ct of 34.9 ± 0.78 for N1 and 36.7 ± 0.55 for N2 (Figure 6A and Supplementary Figure S3). Our CRISPR/Cas9 fluorescence assay was also able to detect the 1:10,000 dilution above background (Figure 6C). CDC guidelines are for positive tests to have a Ct < 40 [49] and other studies show reduced ability to isolate virus when Ct values exceed 35 [50]. Thus, our fluorescence assay is comparable to qRT-PCR with a LOD that correlates to a Ct of ~35. In contrast, the LOD of LFA was an order of magnitude lower (Figure 6E), which is in keeping with its detection by visualization vs fluorescence, making it less sensitive, and may result in an inability to detect patients with low viral titers and makes follow-on confirmation of rapid tests important. To facilitate streamlined confirmation of LFAs, part of our design strategy was to employ a reverse transcription step using oligo dT/random hexamer priming. This allows for the same sample to be tested by LFA, fluorescence, and/or qRT-PCR. This is differential to some ‘one-pot’ approaches, such as RT LAMP; however, using gene-specific priming in this manner, we observed unacceptably high false priming events during PCR (data not shown). Moreover, the use of oligo dT/hexamer priming allows for standard control gene analysis during confirmation testing using nucleic acid amplification tests in accordance with CDC and WHO guidelines. Additionally, our RT strategy will support whole-genome sequencing that will provide further knowledge on coronaviral strain distribution and prevalence [51]. Follow-on confirmation testing is critical for any field-based assay, particularly for our LFA that does not generate/evaluate a human control gene. Rather, the observed assay control band shows proper function of the LFA strips. Future improvements to our approach will include a human control gene that is differentially labeled such that viral target and control genes can be analyzed on the same LFA.

Coronaviruses have genetic proofreading systems [52]; however, mutations occur with potential to confer favorable properties, including increased infectivity or to diminish diagnostic capabilities [53]. S gene mutations such as D614G or N501Y and ORF8a L84S are suggested to have higher rates of infectivity [33,34,35]. The S genes in which the D614 and N501Y residues reside are homologous between SARS-CoV and SARS-CoV-2 [54]. In contrast, the ORF8a gene shows little homology between coronaviruses and the L846 polymorphism is in the seed sequence of the sgRNA using the CRISPR/Cas9 enzyme from *Streptococcus pyogenes* (Figure 2A). The seed sequence is the first ~10 bp proximal to the PAM and dictates specificity to a higher degree than PAM distal sequences [55,56,57,58,59]. These properties make ORF8a a desirable target site for overall specificity and single nucleotide analysis. Using wild-type or a high-fidelity Cas9, we assessed the single nucleotide distinction capabilities of Cas9 nuclease for ORF8a L84 (thymine) or S84 (cytosine) in our fluorometric assay. These data showed a trend for each enzyme in generating higher fluorescence signals when perfect homology between the DNA:sgRNA was present; however, the differences were not statistically significant (Supplementary Figure S6A,B). In contrast, analysis with bdCas9 and LFA allowed for single-nucleotide resolution when a soak DNA was included that was mismatched from the target DNA sgRNA site by 1 bp (Figures S6C–E and S2B, in the Supplementary Materials). Being able to rapidly obtain information on viral polymorphisms could greatly enhance the ability to track the spread or infectivity of novel viral strains. The mutational rate of SARS-CoV-2 shows hotspots in the Orf1ab [60] gene, which may owe to its larger size, and diagnostics that require sequence specificity such as ours may be invalidated should a target site be mutated [53]. However, the plasticity of CRISPR/Cas9 targeting allows for the rapid development and deployment of new reagents to circumvent this. Further, our deliberate use of wholly commercial reagents, most of which are obtained lyophilized and therefore highly stable, supports the development and archival of assays and reagents for current and emergent biological threat events.

In this study, we showed proof-of-principle for Cas9 in detecting target sequences for analysis by LFA or fluorometry. Single nucleotide resolution by LFA can aid in strain identification and has broad applicability for rapidly assessing circulating viral pathogens or other targets for diagnostics, prognostics, drug metabolism, etc. [61]. Fluorescence-based analysis showed high specificity and sensitivity, and in a multiplex fashion, was able to identify four disparate respiratory viral pathogen sequences. LFA allows for field-based application, while the fluorescence assay is highly scalable, allowing for quicker turnaround times. As others have reported [62], the robust amplification obtained by RPA and LAMP can result in cross-contamination and special precautions are required, particularly in multi-step reaction conditions such as ours. In addition, faint bands on LFA can occur [63], particularly with increased exposure time mandating the inclusion of rigorous controls and readout standard operating procedures (i.e., evaluation in <5 min).

## 5. Conclusions

The approval of the SARS-CoV-2 vaccine is highly promising but the time between the first doses and herd immunity will be months. Using commercial reagents, we describe a Cas-9-based detection methodology for nucleic acid detection using lateral flow assays and fluorescence signal generation. Our approach adds to the armamentarium of testing methodologies that can be brought to bear to bridge the immunization–immunity gap.

## 6. Patents

Patent pending.

## Figures and Tables

**Table 1:** Primers and synthetic templates.

Identification	Sequence (5′-3′)	Function
SARS-Co-V2 FAM Forward	/56-FAM/GAATTGTGCGTGGATGAGGCTGG	FAM/FITC-labeled forward primer for SARS-Co-V2 amplification from synthetic or viral genome templates.
SARS-Co-V2 Biotinylated Reverse	/5Biosg/CAACACGAACGTCATGATACTC	Biotinylated-labeled reverse primer for SARS-Co-V2 amplification from synthetic template.
SARS-Co-V2 Unlabeled Reverse	CAACACGAACGTCATGATACTC	Reverse primer for SARS-Co-V2 amplification from synthetic template.
SARS-Co-V2 Genome Reverse	TTAGATGAAATCTAAAACAACACG	Reverse primer for SARS-Co-V2 amplification from genomic RNA template.
SARS-Co-V2 T SNP DNA PCR template.	GAATTGTGCGTGGATGAG GCTGGTTCTAAATCACCCA TTCAGTACATCGATATCGGT AATTATACAGTTTCCTGTTTA CCTTTTACAATTAATTGCCAG GAACCTAAATTGGGTAGTCTT GTAGTGCGTTGTTCGTTCTATG AAGACTTTTTAGAGTATCATG ACGTTCGTGTTG	Synthetic SARS-CoV-2 PCR template containing a thymine SNP at position 28144.
SARS-Co-V2 C SNP DNA PCR template.	GAATTGTGCGTGGATGAG GCTGGTTCTAAATCACCC ATTCAGTACATCGATATC GGTAATTATACAGTTTCC TGTTCACCTTTTACAATTA ATTGCCAGGAACCTAAAT TGGGTAGTCTTGTAGTGCG TTGTTCGTTCTATGAAGACT TTTTAGAGTATCATGACGTTCGTGTTG	Synthetic SARS-CoV-2 PCR template containing a cytosine SNP at position 28144.
Influenza A Forward	CAAGACCAATCYTGTCACCTCTGAC	Forward primer for Influenza A amplification from synthetic DNA.
Influenza A Reverse	GCATTYTGGACAAAVCGTCTACG	Reverse primer for Influenza A amplification from synthetic DNA.
Synthetic Influenza A DNA PCR template	GCTCTCATGGAATGGCTAAAGACA AGACCAATCTTGTCACCTCTGACTA AGGGGATTTTAGGATTTGTGTTCAC GCTCACCGTGCCCAGTGAGCGAGG ACTGCAGCGTAGACGCTTTGTCCAA AATGCCCTAAATGGGAATGGGGAC CCGAACAACATGG	Synthetic PCR template to generate Influenza A DNA amplicons.
Influenza B Forward	TCCTCAAYTCACTCTTCGAGCG	Forward primer for Influenza B amplification from synthetic DNA.
Influenza B Reverse	CGGTGCTCTTGACCAAATTGG	Reverse primer for Influenza B amplification from synthetic DNA.
Synthetic Influenza B DNA PCR template	TACAGTGGAGGATGAAGAAGATGG CCATCGGATCCTCAATTCACTCTTC GAGCGTCTTAATGAAGGACATTCA AAGCCAATTCGAGCAGCTGAAACT GCGGTGGGAGTCTTATCCCAATTTG GTCAAGAGCACCGACTATCACCAG AAGAGGGAGACAAT	Synthetic PCR template to generate Influenza B DNA amplicons.
RSV Forward	GGCAAATATGGAAACATACGTGAA	Forward primer for RSV amplification from synthetic DNA.
RSV Reverse	CATGGGCACCCATATTGTAA	Reverse primer for RSV from synthetic DNA.
Synthetic RSV DNA PCR template	GGCAAATATGGAAACATACGTG AACAAGCTTCACGAAGGCTCCA CATACACAGCTGCTGTTCAATAC AATGTCCTAGAAAAAGACGATG ACCCTGCATCACTTACAATATGG GTGCCCATGTTCCAATCA	Synthetic PCR template to generate RSV DNA amplicons.

FAM = 6-Carboxyfluorescein; 5Biosg = 5′biotin; SNP = single nucleotide polymorphism.

**Table 2:** Fluorescent assay nucleic acids.

Identification	Sequence (5′-3′)	Function
COVID T Probe:	/56-FAM/TCCTGTTTACCTTTTA CAATTAATTGCCAGGA/3IABkFQ/	FAM fluorescent probe with Iowa Black quencher that recognizes SARS-CoV-2 with a thymine at position 28144.
COVID C Probe:	/56-FAM/TCCTGTTCACCTTTTA CAATTAATTGCCAGGA/3IABkFQ	FAM fluorescent probe with Iowa Black quencher that recognizes SARS-CoV-2 with a cytosine at position 28144.
Influenza A Probe:	/5TexRd-XN/CAGTCCTCGCTCACT GGGCACGGTGAGCGTGA/3IAbRQSp/	Texas Red fluorescent probe with Iowa Black quencher that recognizes Influenza A sequence.
Influenza B Probe:	/5YAkYel/TCCCACCGCAGTTTCAG CTGCTCGAATTGGCT/3IABkFQ/	Yakima Yellow fluorescent probe with Iowa Black quencher that recognizes Influenza B sequence.
RSV Probe:	/5Cy3/GCTCCACATACACAGCTG CTGTTCAATACAAT/3IAbRQSp/	Cy3 fluorescent probe with Iowa Black quencher that recognizes RSV sequence.
RSV Probe:	/56-TAMN/GCTCCACATACACAGCTG CTGTTCAATACAAT/3IAbRQSp/	TAMRA fluorescent probe with Iowa Black quencher that recognizes RSV sequence.

**Table 3:** Lateral flow assay soak oligonucleotides.

Identification	Sequence (5′-3′)	Function
PAM Soak Forward:	CGGGAGGGTGGGCGGGAGGGTGGG CGGGAGGGTGGGCGGGAGGGTGGG	PAM-rich ODN that acts as a bait for promiscuous Cas9 binding; sense/top/forward/strand.
PAM Soak Reverse:	CCCACCCTCCCGCCCACCCTCCCG CCCACCCTCCCGCCCACCCTCCCG	PAM-rich ODN that acts as a bait for promiscuous Cas9 binding; anti-sense/bottom/reverse/strand.
C Soak Forward:	GGAGGGTGGGGATTAATTGTAAA AGGTGAACGGGCGGGAGGGTGG	Bait ODN that contains SARS-CoV-2 sequences and a cytosine at position 28144; sense/top/forward/strand. In yellow is the anti-parralel guanine.
C Soak Reverse:	CCACCCTCCCGCCCGTTCACCTTT TACAATTAATCCCCACCCTCC	Bait ODN that contains SARS-CoV-2 sequences and a cytosine at position 28144; anti-sense/bottom/reverse/strand.
T Soak Forward:	GGAGGGTGGGGATTAATTGTAAA AGGTAAACGGGCGGGAGGGTGG	Bait ODN that contains SARS-CoV-2 sequences and a thymine at position 28144; sense/top/forward/strand. In yellow is the anti-parralel adenine.
T Soak Reverse:	CCACCCTCCCGCCCGTTTACCTTT TACAATTAATCCCCACCCTCC	Bait ODN that contains SARS-CoV-2 sequences and a thymine at position 28144; anti-sense/bottom/reverse/strand.

**Table 4:** Single guide RNA sequences.

Identification.	Sequence (5′-3′)	Function
COVID-19 T sgRNA:	AUUAAUUGUAAAAGGUAAAC	Recognizes ORF8a that has a thymine at nucelotide position 28144.
COVID-19 C sgRNA:	AUUAAUUGUAAAAGGUGAAC	Recognizes ORF8a that has a cytosine at nucelotide position 28144.
Influenza A sgRNA:	CUCACCGUGCCCAGUGAGCG	Recognizes the influenza A amplicon.
Influenza B sgRNA:	AAUUCGAGCAGCUGAAACUG	Recognizes the influenza B amplicon.
RSV sgRNA:	UUGAACAGCAGCUGUGUAUG	Recognizes the influenza RSV amplicon.
Control sgRNA:	CACUGGCUGUCGCUUCUCAA	Irrelevant control sgRNA that has no homology to viral genomes.

## Data Availability

Data can be requested from the corresponding author.

## Supplementary Materials

The following are available online at https://www.mdpi.com/2306-5354/8/2/23/s1. Figure S1. Tuning primer concentrations for detection of LFA signals. (**A**) PCR primers labeled with FITC (forward) or biotin (reverse) were designed for amplification of SARS-CoV-2 DNA. (**B**) Amplification was performed with the indicated concentrations of primer in the absence or presence of template and analyzed by LFA. (**C**) Agarose gel analysis of primer concentrations employed in (B). Figure S2. Nucleic acid sequences for sgRNAs and soak DNA. (**A**) The SARS-Co-V2 sgRNA sequences are shown (5′-3′) with the L84S SNP target bases corresponding to SARS-CoV-2 nucleotide position 28144 (shown in red). (**B**) Soak DNA sequences. The PAM-rich soak DNA is rich in GGG trinucleotide PAM sequences. The ORF8a S84 C and L84 T SNP soak sequences are shown with the sgRNA binding site underlined and the respective SNP indicated in red. (**C**). Irrelevant DNA is not bound by COVID gRNA. An irrelevant DNA labeled with FITC was incubated with a perfectly matched sgRNA or the COVID-19 sgRNA. Figure S3. Quantitative reverse transcriptase PCR of coronaviral genomic RNA. RNA from the USA-WA1/2020 strain was diluted from 1:10 to 1:10,000, reverse transcribed, and analyzed using the CDCN gene primer:probe sets. Data are duplicates representative of three analyses and (**A**) is the N1 probe and (**B**) is the N2 probe. The y-axis shows Rn that is the reporter fluorescent signal normalized to ROX and the x-axis shows cycle number. Figure S4. Optimization of LFA for SARS-Co-V2 genomic RNA. DNA amplicons were incubated with Cas9 and a mismatched or COVID-19-specific sgRNA in the presence of various amounts of soak DNA. LFA products are shown with the blue arrow. Figure S5. Correlative genome analysis of SARS-Co-V2 variants. (**A**) The correlation between the D614G (nucleotide 1842) strain of SARS-Co-V2 and strain with L84S due to a single-nucleotide polymorphism at genome nucleotide coordinate 28114 was assessed for the Midwestern state of Minnesota (USA). A pie graph for 1610 patients shows the relationship between the amino acid aspartic acid (D) or glycine (G) at amino acid position 614 and cytosine (C) or thymine (T) nucleotide at 28114. (**B**) The relationship between the ORF8a L84 or S84 and N501Y is shown. The genomes were analyzed from 1816 patients in the United Kingdom. Figure S6. Single-nucleotide resolution of a SARS-Co-V2 variant. (**A**, **B**) Comparison of wild-type Cas9 (**A**) and SpyFi™ Cas9 (**B**) for single-nucleotide recognition. COVID-19 DNA amplicons with a thymine at position 28114 were interrogated with a sgRNA either perfectly matched (T sgRNA) or mismatched by a single base pair (C sgRNA). The fluorescence values for three independent experiments for (**A**) wild-type Cas9 and (**B**) SpyFi™ Cas9 are shown as mean and standard deviation. Controls were an unmatched sgRNA with the indicated Cas9 and probe:DNA hybridization products with no addition of Cas9. Three independent experiments in duplicate were performed with both Cas9 and SpyFi™ included on the same 96-well assay plate. (**C**–**E**) Single nucleotide detection via LFA. (C) Experimental schema. SARS-Co-V2 DNA amplicons with a thymine (T SNP) or cytosine (C SNP) at ORF8a position 28114 were amplified using a FITC-labeled primer. bdCas9 was complexed with a perfectly matched or single base pair mismatched sgRNA. Decoy soak DNA was included as a PAM-rich soak or was mismatched by a single base pair to the specific sgRNA complexed with bdCas9. Dashed lines indicate the blockade of Cas9 by soak DNA. COVID target DNA with a thymine (**D**) or cytosine (**E**) were interrogated with the sgRNAs shown under the LFA test strips. Soak refers to whether the reaction contained a decoy DNA with a cytosine (**D**), thymine (**E**), or the PAM-rich soak DNA. Data are representative of five independent experiments and the blue and black arrows represent assay control and test bands, respectively.
