# Supplementary material for: CRISPR/Cas9-Based Lateral Flow and Fluorescence Diagnostics
Source: Bioengineering (Basel). 2021 Feb 12;8(2):23. doi: 10.3390/bioengineering8020023 (PMC7918862; doi:10.3390/bioengineering8020023)
Supplement: Supplementary file 1 [file bioengineering-08-00023-s001.pdf]

bioengineering

MDPI

Article

CRISPR/Cas9-Based Lateral Flow and Fluorescence Diagnostics

Mark J. Osborn [1,*], Akshay Bhardwaj [1], Samuel P. Bingea [1], Friederike Knipping [1], Colby J. Feser [1], Christopher J. Lees [1], Daniel P. Collins [2], Clifford J. Steer [3,4], Bruce R. Blazar [1] and Jakub Tolar [1]

[1] Medical School, Department of Pediatrics, Division of Blood and Marrow Transplant, University of Minnesota, Minneapolis, MN 55455, USA; bhard009@umn.edu (A.B.); binge042@umn.edu (S.P.B.); fknippin@umn.edu (F.K.); feser004@umn.edu (C.J.F.); leesx002@umn.edu (C.J.L.); blaza001@umn.edu (B.R.B.); tolar003@umn.edu (J.T.)
[2] Cytomedical Design Group, LLC, Saint Paul, MN 55127, USA; dc@cmdgllc.com
[3] Department of Medicine, University of Minnesota Medical School, Minneapolis, MN 55455, USA; steer001@umn.edu
[4] Department of Genetics, Cell Biology and Development, University of Minnesota, Medical School, Minneapolis, MN 55455, USA
* Correspondence: osbor026@umn.edu; Tel.: +1-612-625-7609

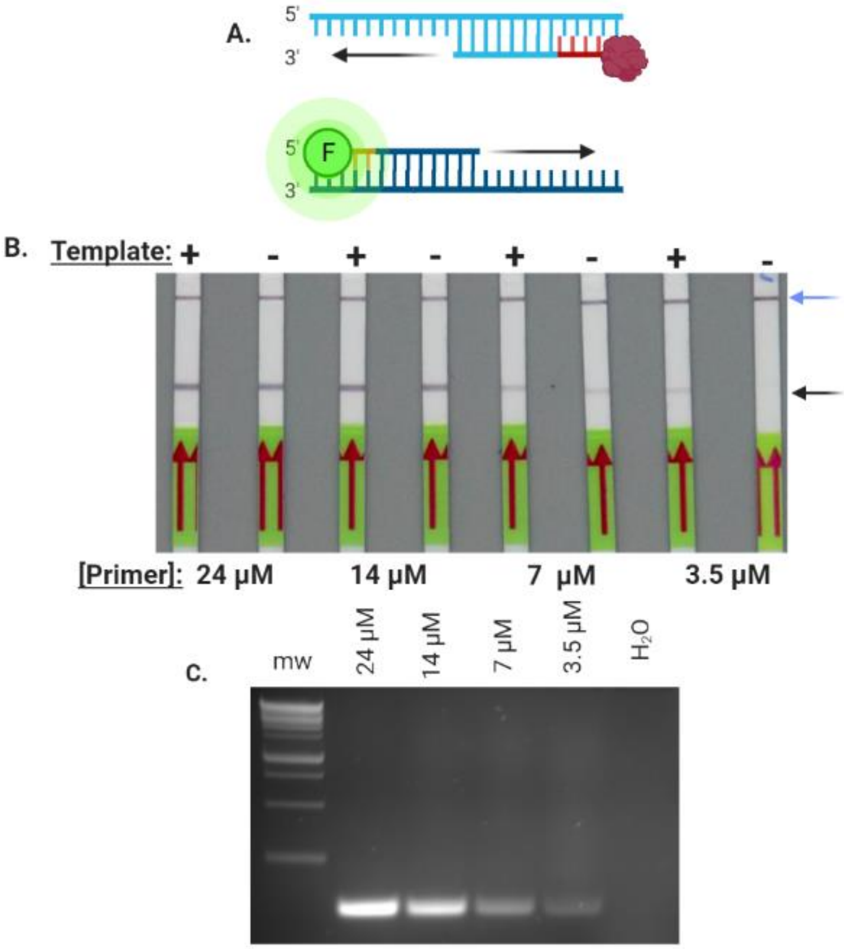

Figure S1. Tuning primer concentrations for detection of LFA signals. (**A**) PCR primers labeled with FITC (forward) or biotin (reverse) were designed for amplification of SARS-CoV-2 DNA. (**B**) Amplification was performed with the indicated concentrations of primer in the absence or presence of template and analyzed by LFA. (**C**) Agarose gel analysis of primer concentrations employed in (B).

Publisher's Note: MDPI stays neutral with regard to jurisdictional claims in published maps and institutional affiliations.

Copyright: © 2021 by the authors. Submitted for possible open access publication under the terms and conditions of the Creative Commons Attribution (CC BY) license (http://creativecommons.org/licenses/by/4.0/).

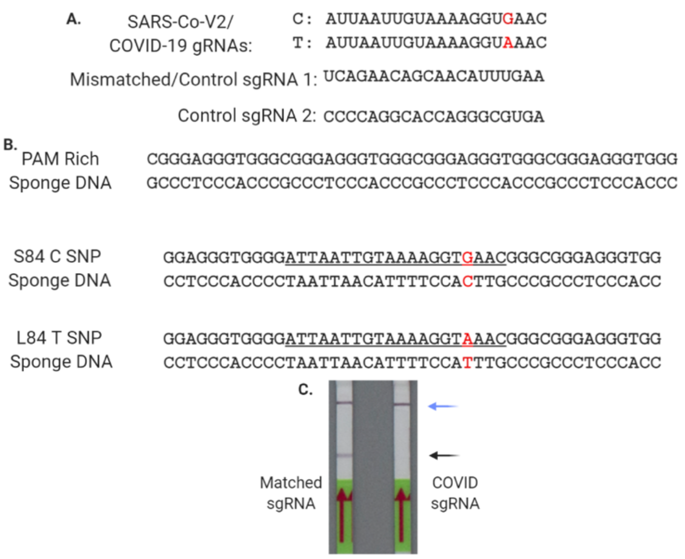

Figure S2. Nucleic acid sequences for sgRNAs and soak DNA. (**A**) The SARS-Co-V2 sgRNA sequences are shown (5′-3′) with the L84S SNP target bases corresponding to SARS-CoV-2 nucleotide position 28144 (shown in red). (**B**) Soak DNA sequences. The PAM-rich soak DNA is rich in GGG trinucleotide PAM sequences. The ORF8a S84 C and L84 T SNP soak sequences are shown with the sgRNA binding site underlined and the respective SNP indicated in red. (**C**). Irrelevant DNA is not bound by COVID gRNA. An irrelevant DNA labeled with FITC was incubated with a perfectly matched sgRNA or the COVID-19 sgRNA.

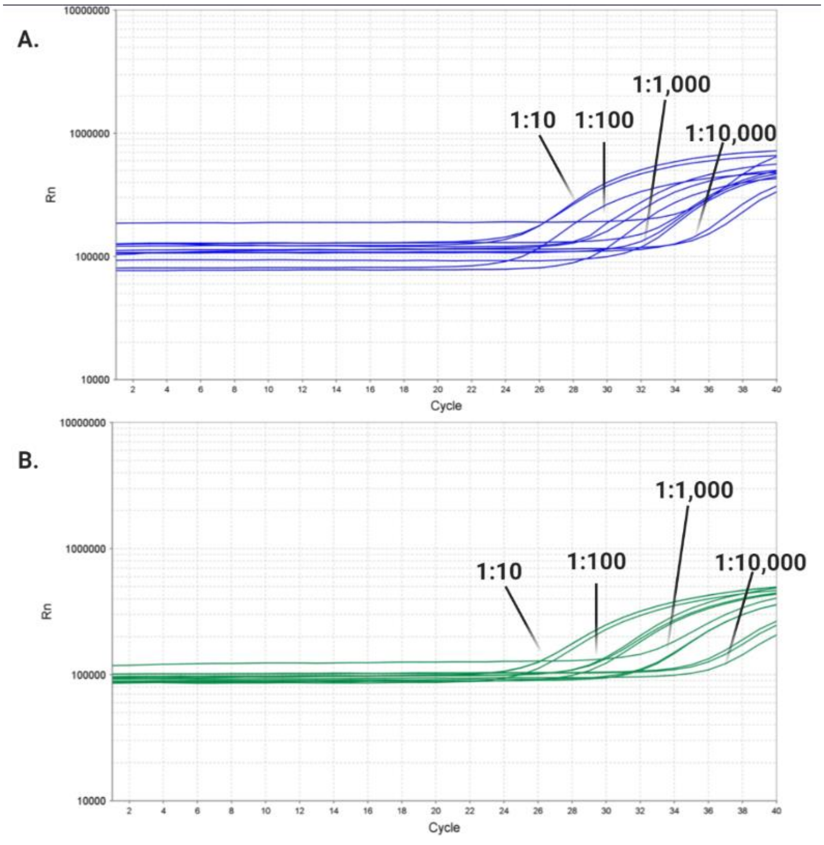

Figure S3. Quantitative reverse transcriptase PCR of coronaviral genomic RNA. RNA from the USA-WA1/2020 strain was diluted from 1:10 to 1:10,000, reverse transcribed, and analyzed using the CDCN gene primer:probe sets. Data are duplicates representative of three analyses and (**A**) is the N1 probe and (**B**) is the N2 probe. The y-axis shows Rn that is the reporter fluorescent signal normalized to ROX and the x-axis shows cycle number.

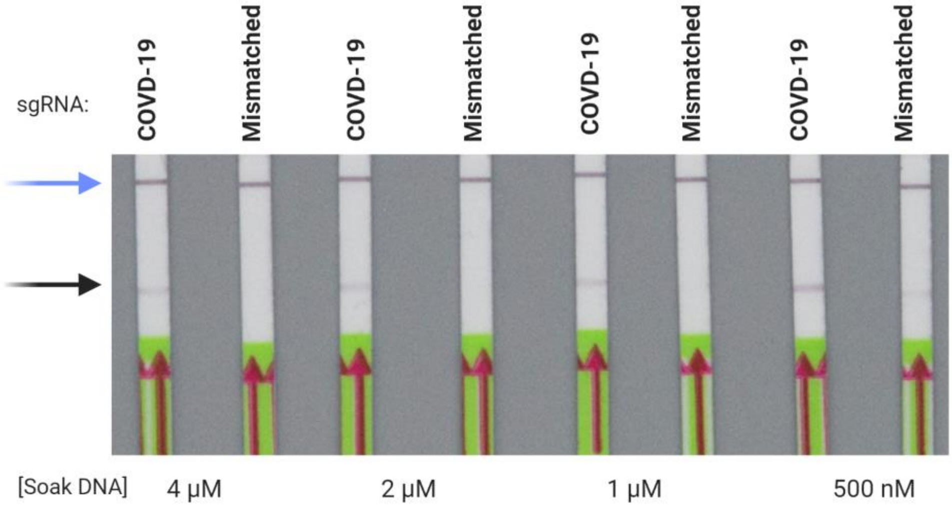

Figure S4. Optimization of LFA for SARS-Co-V2 genomic RNA. DNA amplicons were incubated with Cas9 and a mismatched or COVID-19-specific sgRNA in the presence of various amounts of soak DNA. LFA products are shown with the blue arrow.

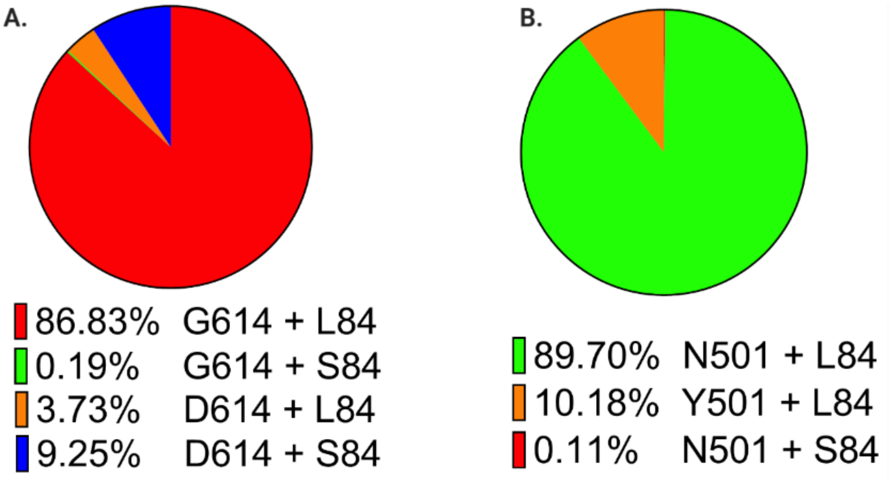

Figure S5. Correlative genome analysis of SARS-Co-V2 variants. (**A**) The correlation between the D614G (nucleotide 1842) strain of SARS-Co-V2 and strain with L84S due to a single-nucleotide polymorphism at genome nucleotide coordinate 28114 was assessed for the Midwestern state of Minnesota (USA). A pie graph for 1610 patients shows the relationship between the amino acid aspartic acid (D) or glycine (G) at amino acid position 614 and cytosine (C) or thymine (T) nucleotide at 28114. (**B**) The relationship between the ORF8a L84 or S84 and N501Y is shown. The genomes were analyzed from 1816 patients in the United Kingdom.

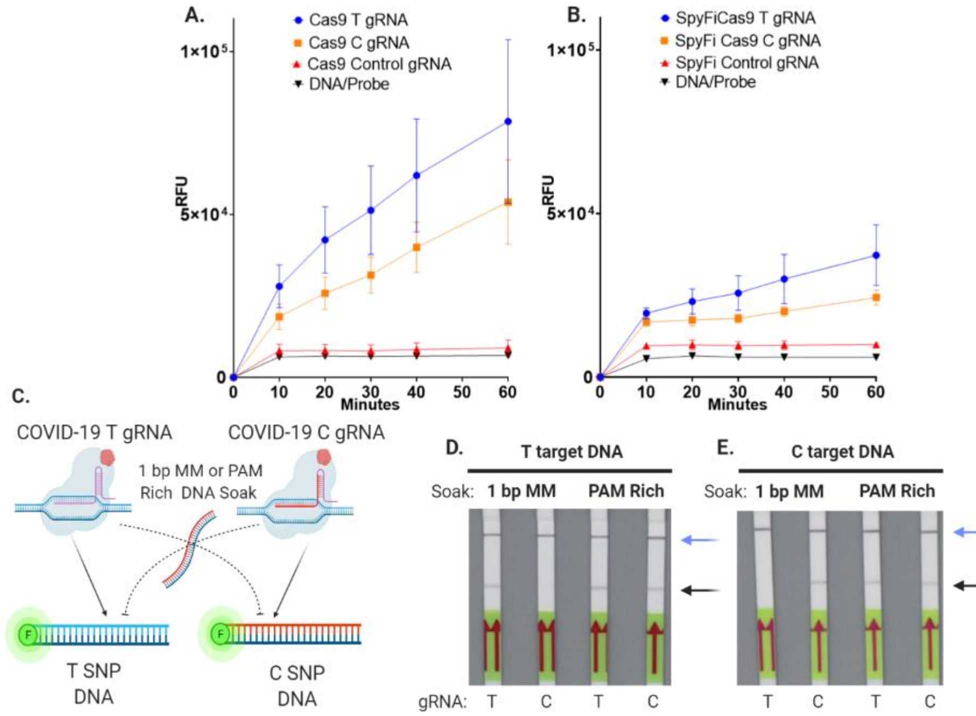

Figure S6. Single-nucleotide resolution of a SARS-Co-V2 variant. (**A**, **B**) Comparison of wild-type Cas9 (**A**) and SpyFi™ Cas9 (**B**) for single-nucleotide recognition. COVID-19 DNA amplicons with a thymine at position 28114 were interrogated with a sgRNA either perfectly matched (T sgRNA) or mismatched by a single base pair (C sgRNA). The fluorescence values for three independent experiments for (**A**) wild-type Cas9 and (**B**) SpyFi™ Cas9 are shown as mean and standard deviation. Controls were an unmatched sgRNA with the indicated Cas9 and probe:DNA hybridization products with no addition of Cas9. Three independent experiments in duplicate were performed with both Cas9 and SpyFi™ included on the same 96-well assay plate. (**C**–**E**) Single nucleotide detection via LFA. (**C**) Experimental schema. SARS-Co-V2 DNA amplicons with a thymine (T SNP) or cytosine (C SNP) at ORF8a position 28114 were amplified using a FITC-labeled primer. bdCas9 was complexed with a perfectly matched or single base pair mismatched sgRNA. Decoy soak DNA was included as a PAM-rich soak or was mismatched by a single base pair to the specific sgRNA complexed with bdCas9. Dashed lines indicate the blockade of Cas9 by soak DNA. COVID target DNA with a thymine (**D**) or cytosine (**E**) were interrogated with the sgRNAs shown under the LFA test strips. Soak refers to whether the reaction contained a decoy DNA with a cytosine (**D**), thymine (**E**), or the PAM-rich soak DNA. Data are representative of five independent experiments and the blue and black arrows represent assay control and test bands, respectively.
